# Thermal pretreatment facilitates recovery from prolonged low‐frequency force depression in rat fast‐twitch muscle

**DOI:** 10.14814/phy2.13853

**Published:** 2018-09-02

**Authors:** Daiki Watanabe, Chihiro Aibara, Naoki Okada, Masanobu Wada

**Affiliations:** ^1^ Graduate School of Integrated Arts and Sciences Hiroshima University Hiroshima Japan; ^2^ Research Fellow of Japan Society for the Promotion of Science Tokyo Japan

**Keywords:** Calcium release, low‐frequency muscle fatigue, reactive oxygen species, sarcoplasmic reticulum

## Abstract

The aim of this study was to examine whether thermal pretreatment can accelerate recovery from prolonged low‐frequency force depression. The hindlimbs of thermal treated (T‐treated) rats were immersed in water heated to 42.0°C for 20 min (thermal pretreatment). The thermal pretreatment was performed once a day for 5 days before fatiguing stimulation. Intact gastrocnemius muscles were electrically stimulated via the sciatic nerve until force was reduced to ~50% of the initial and dissected immediately [recovery 0 (REC0)] or 60 min [recovery 60 (REC60)] following the cessation of stimulation. Using skinned fiber prepared from the superficial region, the ratio of force at 1 Hz to that at 50 Hz (low‐to‐high force ratio), the ratio of depolarization (depol)‐induced force to maximum Ca^2+^‐activated force (depol/max Ca^2+^ force ratio), the steepness of force‐Ca^2+^ concentration curves, and myofibrillar Ca^2+^ sensitivity were measured. At REC0, the low‐to‐high force ratio and depol/max Ca^2+^ force ratio decreased in stimulated muscles from both non‐ and thermal‐treated rats. At REC60, these two parameters remained depressed in non‐treated rats, whereas they reverted to resting levels in T‐treated rats. Thermal pretreatment exerted no effect on myofibrillar Ca^2+^ sensitivity. The present results reveal that thermal pretreatment can facilitate recovery of submaximum force after vigorous contraction, which is mediated via a quick return of Ca^2+^ release from the sarcoplasmic reticulum to resting levels.

## Introduction

Vigorous muscle activity eventually leads to fatigue development with decreased force production and slower contractions. A specific type of delayed force recovery was originally observed by Edwards et al. (1977) in human adductor pollicis muscles. In this type of muscle fatigue, tetanic force is more depressed at low than at high stimulation frequencies, which is now called “prolonged low‐frequency force depression (PLFFD)” (Bruton et al. 2008; Watanabe and Wada 2016). It is proposed that decreased myofibrillar Ca^2+^ (my‐Ca^2+^) sensitivity and/or reduced Ca^2+^ release from the sarcoplasmic reticulum (SR) can in theory mediate depressions in force at low frequencies with little influence at high frequencies (Wada et al. 2013). In the skeletal muscle, reactive oxygen and nitrogen species (ROS/RNS) are moderately generated at rest, and their production increases markedly with intense and prolonged contractions. Increased ROS/RNS have been implicated in changes in both my‐Ca^2+^ sensitivity and SR Ca^2+^ release. For example, accumulation of superoxide and hydroxyl radical accounts for depressions in SR Ca^2+^ release and my‐Ca^2+^ sensitivity, respectively (Bruton et al. 2008; Murphy et al. 2008). More recently, treatment of muscle fibers with *S*‐nitrosylation was reported to bring about decreased my‐Ca^2+^ sensitivity (Dutka et al. 2017).

As pointed out by Ørtenblad and Stephenson (2003), muscle fatigue may play an important role in protecting the muscle cell against irreversible damage. However, in some circumstances (e.g., successive days of competitive sport), one would expect that a loss of muscle performance, which results from the preceding muscle activities, is minimized and diminished performance is rapidly restored. To this end, various pretreatment strategies have been performed to date, depending on environmental conditions, exercise intensities, and contraction types. These include precooling (Katica et al. 2018), chronic exposure to altitude (Kenney et al. 2015), heat acclimation (Kenney et al. 2015), nutritional supplement (Mishima et al. 2008), and thermal treatment (Touchberry et al. 2012).

Of these, thermal pretreatment (i.e., warming muscles in advance) has been shown to exert beneficial effects on muscle, such as increased protein synthesis (Kojima et al. 2007), blunting of muscle damage (Touchberry et al. 2012), and inhibition of muscle atrophy (Tamura et al. 2015). Additionally, a recent work by Tamura et al. (2015) has revealed that thermal pretreatment can ameliorate denervation‐dependent oxidative stress. This finding is of special interest in view of a cause of PLFFD and raises the plausible hypothesis that the extent of PLFFD that occurs after intense muscle contraction would be attenuated by thermal pretreatment. However, no studies have explored this point to date.

The present study was undertaken in order to examine whether thermal pretreatment can improve muscle function after fatiguing contraction. One of methods used in many of previous studies on PLFFD is an approach in which isolated muscle fibers are fatigued by direct electrical stimulation (Bruton et al. 2008). Such an approach has some advantages, including a precise control of the fatigue extent and a simultaneous monitoring of the myoplasmic Ca^2+^ concentration ([Ca^2+^]) and force output. It has been pointed out, however, that the results obtained from isolated muscles may not necessarily reflect changes in vivo (see DISCUSSION). To gain insights into phenomena that occur in living muscles, this study utilized an in vivo model for muscle fatigue, developed by our laboratory (Watanabe and Wada 2016; Kanzaki et al. 2018).

## Methods

### Ethical approval and animal care

All experimental procedures used in this study were approved by the Animal Care Committee of Hiroshima University. Male Wistar rats (*N* = 32) aged 10 weeks were used in this study. The animals were provided with water and standard chow (CE‐2; CLEA Japan, Tokyo, Japan) ad libitum and were individually housed in a cage in a thermally controlled room at 20–24°C with 12‐h light/dark cycle. An intraperitoneal injection of a mixture of medetomidine (0.4 mg kg‐body wt^−1^), midazolam (2.0 mg kg‐body wt^−1^), and butorphanol (2.5 mg kg body wt^−1^) was used for anesthesia. At the end of the experiments, the rats were euthanized with an overdose of pentobarbital sodium (200 mg kg body wt^−1^), followed by cervical dislocation.

### Thermal pretreatment and fatiguing stimulation

The rats were randomly divided a thermal treated (T‐treated) and a non‐treated group (*N* = 16 for each group). Under anesthesia, the hindlimbs of T‐treated rats were immersed in water heated to 42.0°C for 20 min. Thermal pretreatment started 5 days prior to fatiguing stimulation (see below) and was performed once a day, five times in total. Non‐treated rats were anesthetized without thermal treatment.

The fatiguing stimulation protocol was performed 24 h after the last thermal pretreatment. Intact gastrocnemius (GAS) muscles from the left hindlimb was electrically stimulated in vivo via the sciatic nerve, as described previously (Watanabe and Wada 2016). The animals were placed in a supine position and the left hindlimb was attached to a homemade foot holder connected with an isometric transducer. The sciatic nerve was then stimulated (70 Hz, 0.35‐sec train, every 2 sec) until the force was reduced to ~50% of the initial force (fatiguing stimulation). Immediately [recovery 0 (REC0)] or 60 min [recovery 60 (REC60)] after fatiguing stimulation, the GAS muscles of stimulated (left) and rested (right) legs were excised. The superficial region of the GAS muscle was used for analyses. It has been previously shown that this region of rat GAS muscle is predominantly composed of type IIB fibers (~90%), with small amounts of type IIX fibers (Pette and Staron 1990).

### Skinned fiber preparation

Mechanically skinned fibers were prepared as described by Watanabe and Wada (2016). A part of the excised GAS muscle was pinned out at resting length under paraffin oil and was kept cool on an ice pack. Single muscle fibers were dissected under a stereomicroscope and were mechanically skinned by rolling back the sarcolemma with fine forceps. A segment of the skinned fiber was connected to a force transducer (Muscle tester, SI, Germany), stretched to 1.2 times resting length, and transferred to a bath containing K‐hexamethylene‐diaminetetraacetic acid (HDTA) solution (see below) to equilibrate. Although the GAS muscle was placed in cold paraffin oil immediately after its excision, it took several minutes (5–20 min) to start measurements on skinned fibers. In our preliminary experiment, we ascertained the time‐dependent effect of immersion of GAS muscles in cold paraffin oil and observed that fatiguing stimulation‐induced changes in the contractile properties of skinned fibers persisted at least up to 50 min after placing the GAS muscle in paraffin oil.

### Solutions

All solutions for skinned fiber experiments were prepared as described in detail elsewhere (Watanabe et al. 2015). The solutions were basically composed of 36 mmol/L Na^+^, 126 mmol/L K^+^, 90 mmol/L *N*‐2‐hydroxyethylpiperazine‐*N*’‐2‐ethanesulfonic acid, 8 mmol/L ATP_total_, and 10 mmol/L creatine phosphate (pH 7.09–7.11 at 25°C. The free Mg^2+^ concentration was set at 1.0 mmol/L. The K‐HDTA solution additionally contained 50 mmol/L HDTA and 0.05 mmol/L EGTA to give 10^−6.9^ mol/L free [Ca^2+^]. Na‐HDTA solution was similar to the K‐HDTA solution, with all K^+^ replaced with Na^+^. The maximum (max) Ca^2+^ activation solution was also similar to the K‐HDTA solution, but with all HDTA replaced with 49.5 mmol/L Ca‐EGTA and 0.5 mmol/L free EGTA to heavily buffer the free [Ca^2+^] at 10^−4.7^ mol/L. A similar strongly EGTA‐buffered solution containing 50 mmol/L EGTA (<10^−9^ mol/L) and no Ca‐EGTA was used as a relaxing solution. These two solutions were mixed in an appropriate ratio with free [Ca^2+^] in the range of 10^−9^–10^−4.7^ mol/L and used for force–[Ca^2+^] relationship analysis.

### Na^+^ depolarization‐induced force

After 2 min of immersion in the K‐HDTA solution, the fiber was depolarized by replacing the K‐HDTA with the Na‐HDTA solution, which results in Ca^2+^ release from the SR and substantial force response, and was then returned to the K‐HDTA solution for 2 min. This procedure was repeated until the peak force responses were stable. The fiber was then exposed to the max Ca^2+^ activation solution. Depolarization (depol)‐induced force was evaluated relative to max Ca^2+^‐activated force (depol/max Ca^2+^ force ratio). Previous studies (Watanabe and Wada 2016) have indicated that the depol/max Ca^2+^ force ratio can be used as an indicator of SR Ca^2+^ release.

### Action potential stimulation‐induced and specific maximum Ca^2+^‐activated force

The skinned fiber was placed in a small chamber containing ~500 *μ*L of K‐HDTA solution and was centered between two platinum electrodes. The fiber length was adjusted to optimize the force developed at 50 Hz. The fiber was stimulated with 1‐msec pulses at 80 V cm^−1^ with a single pulse (for twitch) and 20 pulses at 50 Hz and then exposed to the max Ca^2+^ activation solution. Max Ca^2+^‐activated force was normalized to the cross‐sectional area of the fibers (specific max Ca^2+^ force). The cross‐sectional area was modeled as a circular profile if the diameter was similar along the fiber segment and was calculated from an average of three width measurements.

### Steepness of force–[Ca^2+^] curves and myofibrillar Ca^2+^ sensitivity

The force–Ca^2+^ relationship was determined by exposing the skinned fiber to a sequence of solutions heavily buffered at progressively higher free [Ca^2+^] (10^−9.0^–10^−4.7^ mol/L). The force response produced at each [Ca^2+^] was expressed as a percentage of the max force. Using SigmaPlot 14 software (HULINKS, Tokyo, Japan), Hill coefficient (*n*_H_) and [Ca^2+^]_50_ ([Ca^2+^] at half‐maximum force) were calculated. The steepness of force‐[Ca^2+^] curves and my‐Ca^2+^ sensitivity were evaluated on the basis of *n*_H_ and [Ca^2+^]_50_, respectively.

### Immunoblot

After various stressful stimuli, heat shock proteins (HSPs) are upregulated and interact with many proteins to help preserve and restore their function (Oishi et al. 2002; Mymrikov et al. 2011). It has been previously shown that of three HSPs (i.e., *α*B‐crystallin, HSP25, and HSP70) present in rat skeletal muscles, HSP70 (also known as HSP72) is highly susceptible to heat stress (Tamura et al. 2015). To elucidate whether HSPs are involved in thermal treatment‐induced changes (see below), the amounts of HSP70 were analyzed by immunoblot.

Frozen muscles were pulverized under liquid N_2_ in a steel mortar. The resultant muscle powder was diluted in 9 volumes (vol mass^−1^) of ice‐cold homogenizing buffer composed of 10 mmol/L Tris/HCl (pH 7.0), 150 mmol/L NaCl, 5 mmol/L NaF, 1 mmol/L Na‐orthovanadate, 1% (vol vol^−1^) Triton X‐100, 22 *μ*mol/L leupeptin, and 14 *μ*mol/L pepstatin and was homogenized on ice using a hand‐held homogenizer. The protein content was determined by the Bradford assay using bovine serum albumin as the standard (Bradford 1976). A quantity of 20 *μ*g of proteins were applied to a 10% (mass vol^−1^) polyacrylamide gel and transferred to polyvinylidene difluoride membranes. The membranes were incubated at room temperature with anti‐HSP70 (1:1000 dilution; ab2787, Abcam) and glyceraldehyde‐3‐phosphate dehydrogenase (GAPDH; 1:10000 dilution; 5A12, Wako). The membranes were then incubated with secondary antibodies (1:5000 dilution, 172‐1011, BioRad) at room temperature. Immunoreactive bands were visualized with chemiluminescence reagent (GE Healthcare, Pittsburgh, PA) and evaluated using ImageJ software (National Institutes of Health, Bethesda, MD). The HSP70 amounts were evaluated relative to the band density of GAPDH.

### Statistical analyses

Data are presented as means + SD. In this study, the effect of recovery time was not investigated. Two‐way ANOVA was used to determine the effects of thermal treatment and fatiguing stimulation. When significant differences were detected, Holm–Sidak post hoc test was performed. The level of significance was set at *P *<* *0.05.

## Results

### Low‐to‐high force ratio

**Figure 1:**
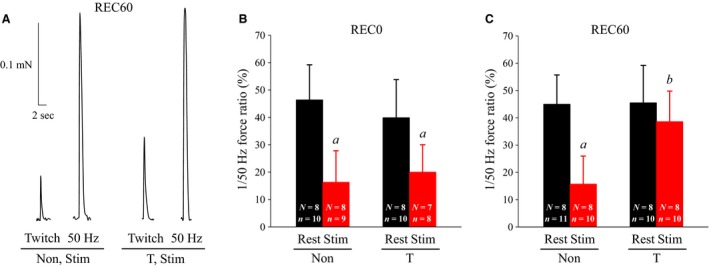
Effects of thermal pretreatment and fatiguing stimulation on low‐to‐high force ratio (1 vs. 50 Hz) in skinned fiber. Hindlimbs of thermal‐treated rats were immersed to 42.0°C for 20 min day^−1^, which was performed five times before fatiguing stimulation. Twenty‐four hours after the last thermal pretreatment, intact gastrocnemius muscle of the left hindlimb was stimulated in vivo until the force was reduced to ~50% of the initial force (fatiguing stimulation). Immediately or 60 min after fatiguing stimulation, skinned fibers were prepared from gastrocnemius muscles. (A) Typical sample of action potential‐induced force response in stimulated (fatigued) fibers from non‐ and thermal‐treated rats that were allowed to rest for 60 min. Action potential‐induced contractions were electrically elicited using a single pulse (for twitch) and 20 pulses at 50 Hz. (B and C) ratio of twitch (1 Hz) force to force at 50 Hz. Values are means + SD. “*n*” denotes number of fibers and “*N*” the number of rats from which the fibers were obtained. ^*a*^ *P* < 0.05, significantly different from rested muscles; ^*b*^ *P* < 0.05, significantly different from stimulated muscles from non‐treated rats. Non, non‐treated; T, thermal‐treated; REC0, recovery 0 min; REC60, recovery 60 min; Stim, stimulated.

Intact GAS muscles were fatigued by repeated stimulation until the force was reduced to ~50% of the initial force. There were no significant differences in the time to fatigue among four groups examined (~90–120 sec). It has been shown previously that in skinned fibers, the ratio of twitch force (force at 1 Hz) to force at 50 Hz (low‐to‐high force ratio) in action potential‐induced forces can be utilized as an indicator of PLFFD (Watanabe et al. 2015; Watanabe and Wada 2016). At REC0, the low‐to‐high force ratio decreased in stimulated fibers to 35% and 50% of that in rested fibers from non‐ and T‐treated rats, respectively (Fig. 1B). At REC60, the ratio remained depressed in non‐treated rats, whereas it reverted to the resting level in T‐treated rats (Fig. 1C).

### Depolarization/maximum Ca^2+^ force ratio

**Figure 2:**
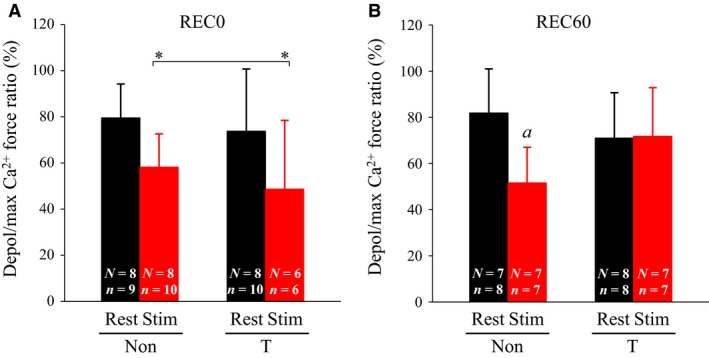
Effects of thermal pretreatment and fatiguing stimulation on depolarization (depol)‐induced force in skinned fiber. For the protocol of thermal pretreatment and fatiguing stimulation, see legend of Figure 1. Depol‐induced force was elicited by replacing the K‐HDTA with the Na‐HDTA solution and expressed as a percentage of maximum Ca^2+^‐activated force (depol/max Ca^2+^ force ratio). Values are means + SD. “*n*” denotes number of fibers and “*N*” the number of rats from which the fibers were obtained. *P <* *0.05, significant main effect for stimulation (rested > stimulated); ^*a*^ *P* < 0.05, significantly different from rested muscles. Non, non‐treated; T, thermal‐treated; REC0, recovery 0 min; REC60, recovery 60 min; Stim, stimulated: HDTA, hexamethylene‐diaminetetraacetic acid.

The results of the depol/max Ca^2+^ force ratio, an indicator of SR Ca^2+^ release, resembled those of the low‐to‐high force ratio (Fig. 2). At REC0, the depol/max Ca^2+^ ratio decreased in stimulated fibers to a level of 72% and 66% of that in rested fibers from non‐ and T‐treated rats, respectively (Fig. 2A). At REC60, the ratio remained depressed in non‐treated rats, whereas it was fully restored in T‐treated rats (Fig. 2B).

### Specific maximum Ca^2+^ force, myofibrillar Ca^2+^ sensitivity, Hill coefficient, and HSP70 amounts

**Figure 3:**
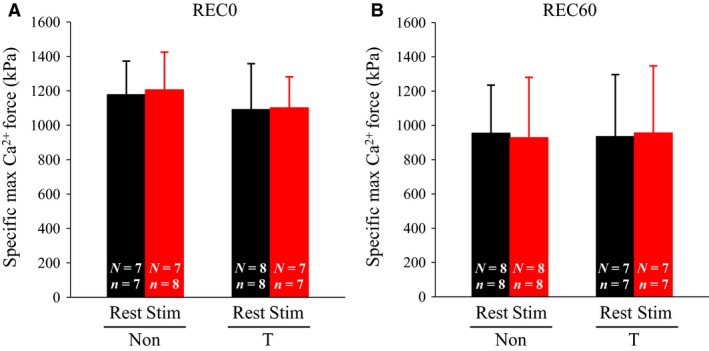
Effects of thermal pretreatment and fatiguing stimulation on specific maximum (max) Ca^2+^ force in skinned fiber. For the protocol of thermal pretreatment and fatiguing stimulation, see legend of Figure 1. Max Ca^2+^‐activated force was elicited by exposing the skinned fiber to heavily buffered Ca^2+^ solution (pCa ~4.7) and normalized to the cross‐sectional area of the fiber. Values are means + SD. “*n*” denotes number of fibers and “*N*” the number of rats from which the fibers were obtained. Non, non‐treated; T, thermal‐treated; REC0, recovery 0 min; REC60, recovery 60 min; Stim, stimulated.

**Figure 4:**
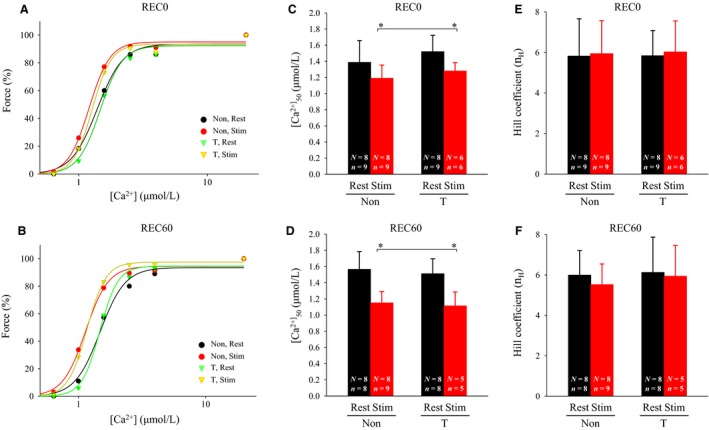
Effects of thermal pretreatment and fatiguing stimulation on myofibrillar Ca^2+^ sensitivity and Hill coefficient in skinned fiber. For the protocol of thermal pretreatment and fatiguing stimulation, see legend of Figure 1. The fiber was exposed to a sequence of solutions heavily buffered at progressively higher free [Ca^2+^] (10^−9.0^–10^−4.7^ mol/L). (A and B) Hill fits to force‐[Ca^2+^] data. The [Ca^2+^] was expressed as a base 10 logarithmic scale. (C and D) mean (+SD) of [Ca^2+^]_50_ for Hill fits in A and B. (E and F) mean (+SD) of Hill coefficient for Hill fits in A and B. “*n*” denotes number of fibers and “*N*” the number of rats from which the fibers were obtained. *P <* *0.05, significant main effect for stimulation (rested > stimulated). Non, non‐treated; T, thermal‐treated; REC0, recovery 0 min; REC60, recovery 60 min; Stim, stimulated; [Ca^2+^], Ca^2+^ concentration; [Ca^2+^]_50_, [Ca^2+^] required for half‐maximum force.

**Figure 5:**
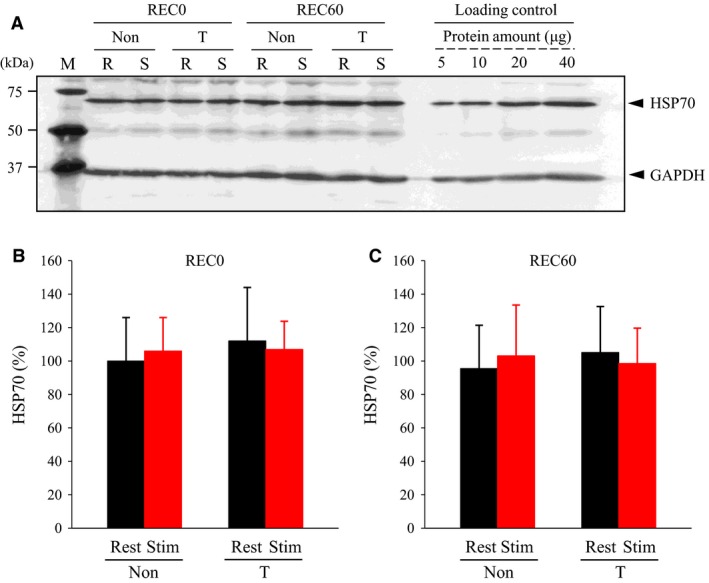
Immunoblot analyses of heat shock protein (HSP) 70. For the protocol of thermal pretreatment and fatiguing stimulation, see legend of Figure 1. (A) immunoblot analyses of HSP70. A quantity of 20 *μ*g of protein was applied to SDS‐PAGE. To ascertain a linear relationship between the band density and the protein amount, 5–40 *μ*g of protein was loaded on the gel for loading control. Molecular mass of makers (M) is indicated in the *left*. (B and C) means + SD of HSP70 amount (*N* = 7 for each muscle). The HSP70 amounts were evaluated relative to the band density of GAPDH. The results are expressed as percentages of the values of rested muscles from non‐treated rats at REC0. Non, non‐treated; T, thermal‐treated; REC0, recovery 0 min; REC60, recovery 60 min; S and Stim, stimulated; R, rested; GAPDH, glyceraldehyde‐3‐phosphate dehydrogenase.

Neither fatiguing stimulation nor thermal treatment resulted in changes in specific max Ca^2+^ force and *n*_H_ at REC0 and REC60 (Figs. 3, 4E, and F). At both REC0 and REC60, my‐Ca^2+^ sensitivity, as measured by [Ca^2+^]_50_, was elevated (i.e., decreased [Ca^2+^]_50_) in stimulated fibers (Fig. 4C and D). At both REC0 and REC60, no significant differences in my‐Ca^2+^ sensitivity were observed between the corresponding fibers from non‐ and T‐treated rats. Inconsistent with the previous findings (Oishi et al. 2002; Touchberry et al. 2012; Tamura et al. 2015), thermal treatment did not evoke increases in the HSP70 amounts (Fig. 5).

## Discussion

To our knowledge, no published study presently exists that shows an effective pretreatment strategy toward overcoming PLFFD. The present investigation, for the first time, provides evidence that thermal pretreatment can accelerate recovery from PLFFD. Skinned fiber analyses reveal that the beneficial effect of thermal treatment results from the faster recovery of impaired function of SR Ca^2+^ release.

Many, if not all (Larkins et al. 2012), studies have revealed that heat stress and/or vigorous muscle contraction result in the upregulation of HPS70 in fast‐ and slow‐twitch muscles from mammals (Oishi et al. 2002; Tupling et al. 2007; Tamura et al. 2015; Hafen et al. 2018). Conversely, the current study showed that neither thermal treatment nor fatiguing stimulation elicited changes in the expression of HSP70 (Fig. 5). The reasons for the conflicting results remain unclear. However, with the consideration that the extent and time of increases in HSP70 differs between studies (Oishi et al. 2002; Touchberry et al. 2012; Tamura et al. 2015; Hafen et al. 2018), it seems likely that differences in thermal treatment protocols used, species examined, and/or muscle volumes yield distinct results. The possibility cannot be excluded that in this study, HSP70 was upregulated at the time other than ~24 h after the last thermal treatment, the only time point investigated, and interacted with some proteins critical for excitation–contraction coupling.

Different ROS are purported to have distinct effects on my‐Ca^2+^ sensitivity and SR Ca^2+^ release, changes that can account for PLFFD (Bruton et al. 2008; Murphy et al. 2008). The chief parent radical species produced in skeletal muscles are superoxide anions, which are converted to hydrogen peroxide via superoxide dismutase (SOD). A single fiber study by Bruton et al. (2008) has revealed that PLFFD is caused by reduced SR Ca^2+^ release in fibers with low SOD activity, but not in fibers with high SOD activity, suggestive that superoxide has the deleterious influence on SR Ca^2+^ release and that reduced SR Ca^2+^ release observed in fatigued fibers (Fig. 2) is likely to involve superoxide.

Based on the inhibitory effect of thermal pretreatment on oxidative stress, which has been reported in previous study (Tamura et al. 2015), we had assumed that thermal pretreatment would mitigate the extent of PLFFD and reductions in SR Ca^2+^ release that occur immediately after fatiguing muscle contraction. However, contrary to our assumption, the present study showed that thermal pretreatment was unable to lessen these two parameters, but instead was able to facilitate recovery of them (Figs. 1 and 2). The mechanisms underlying the influence of thermal pretreatment during recovery presented here are unclear. Provided that superoxide generated during vigorous muscle contraction is implicated (Bruton et al. 2008), some proteins (e.g., ryanodine receptor or dihydropyridine receptor) involved in the regulation of SR Ca^2+^ release would be oxidized, which in turn results in reduced SR Ca^2+^ release. Cysteine and methionine are amino acids that contain sulfhydryl groups and are among the most sensitive to oxidation. Of the cysteine and methionine oxidation products, sulfenic and sulfinic acids and methionine sulfoxide can be reduced back to cysteine and methionine by specific enzymatic systems (Mary et al. 2004). From these findings, thermal pretreatment might be expected to evoke alterations that enhance the function of these systems. This scenario is speculative but an important subject for further research.

My‐Ca^2+^ sensitivity seems likely to be variable depending on the cellular redox. Indeed, it has been demonstrated that my‐Ca^2+^ sensitivity is increased by the exposure of isolated fibers to a low concentration of hydrogen peroxide, while fibers treated with a high concentration display decreased my‐Ca^2+^ sensitivity (Andrade et al. 1998, 2001). Previous studies on isolated muscles have argued that decreased my‐Ca^2+^ sensitivity is one of the causes of PLFFD (Bruton et al. 2008). In experiments using isolated muscles, it is necessary to expose the fibers to supraphysiological oxygen tensions to avoid hypoxia. It is conceivable, therefore, that decreased my‐Ca^2+^ sensitivity found in isolated muscles may be ascribable to severe oxidative stress that rarely occurs in muscles in vivo (Dutka et al. 2012). Contrary to the findings from isolated muscles, the present study using an in vivo model for fatigue showed that fatiguing stimulation resulted in increased my‐Ca^2+^ sensitivity (Fig. 4), which is in accord with our previous observations (Watanabe and Wada 2016). Although not measured in this study, our previous experiments have indicated that in fast‐twitch muscles that are fatigued in vivo, sulfhydryl groups of troponin I are *S*‐glutathionylated, which in turn brings about increased my‐Ca^2+^ sensitivity (Watanabe and Wada 2016). It has been shown that increased *S*‐glutathionylation of troponin I also occurs in exercising human skeletal muscles (Mollica et al. 2012). These findings point out that mammalian fast‐twitch muscles are endowed with a system helping delay onset of muscle fatigue.

The impact of in vitro thermal treatment on the function of the contractile apparatus has been addressed by van der Peol and Stephenson (2002). They found that exposure of rat fast‐twitch muscle to 43°C led to pronounced increases in ROS production, which resulted in reduced specific max Ca^2+^ force and *n*_H_, but the depressions were reversible. It is equivocal as to whether thermal treatment in vivo causes the shift of the intracellular redox state to the oxidized side. However, our results of no changes in specific max Ca^2+^ force and *n*_H_ suggest that if the shift occurs, the influence would disappear at least 24 h after thermal pretreatment.

In summary, we show that thermal pretreatment can accelerate restoration of submaximum force during a recovery period after vigorous contraction, which is mediated via a quick return of the Ca^2+^ release function of SR to resting levels. It is well known that proper physical training not only improves physical performance, but also decreases the risk of developing chronic diseases such as cardiovascular diseases, hypertension, diabetes, and obesity (Kenney et al. 2015). However, the sensation of muscle weakness resulting from PLFFD that occurs with exercise will depress the training efficiency and the motivation of people trying to exercise. The current results suggest that the usage of thermal pretreatment contributes to fast restoration of muscle function following physical training. Additional studies are required to elucidate the mechanism(s) behind the effects of thermal pretreatment on the function of fatigued muscles.

## Conflict of Interest

The authors declare no conflict of interest.
